# Sir Andrew Clark: A Monument

**Published:** 1894-01-20

**Authors:** 


### The Hospital

An Institutional Journal of

The Medical Sciences and Hospital Administration, with

AN EXTRA NURSING SUPPLEMENT.

Vol. XV.—No. 382.] [Jan. 20, 1894.

### Sir Andrew Clark: A Monument.

A great nation like our own is not too ready to raise monuments even to its distinguished children, nor should it be. But the great public, the final arbiter of every man's reputation, is well pleased that some public memorial is to be raised for Sir Andrew Clark. We who are of medicine may feel grateful that the late leader of our profession was recognised by other great leaders as one of themselves. The Premier of England, a Royal duke, the princes of our own and of the venerable Roman Church, the leaders of other great religious organisations, and distinguished members of our own profession, constitute a body of persons who represent in the broadest sense the culture and the conscience of the nation. No man, even when he is dead, may expect to obtain entirely universal approval; but any man, however excellent and however great, might well be satisfied if he knew that within a few weeks of his death and burial the finest flower and fruit of his country's intellect and character had recognised him as of pre-eminent worth. This has proved to be the happy fate of the late Sir Andrew Clark, President of the English College of Physicians.

Certain members of the medical profession have thought that a memorial exclusively medical, not instead of but in addition to the public and general memorial, should have been attempted. Sir Andrew Clark was a great therapeutist of the modern school. He made the attempt to practice medicine scientifically rather than empirically. He conscientiously mastered the details of the new knowledge of physiology, pathology, and the action of remedies which were gradually accumulating in his life-time, and, so far as it was possible for man, he ordered his practice by their dictates and their light. But he was under no absolute obligation to do this; at any rate, during the last fifteen or twenty years of his life. Not only had he made his reputation, but he had proved by his personal experience the practical value of the older methods of treatment; and there is no physician of any experience and intellectual capacity but knows that the older and more purely emperical methods would in ninety-nine of every hundred of his cases have been quite as successful in actual practice as the newer and more scientific. Sir Andrew Clark might therefore, if he had so chosen, have spared himself the arduous labour of keeping himself abreast of the knowledge and newer methods of recent times. But, with the utmost thoroughness and conscientiousness he mastered every new fact and theory as they were brought into notice, and according to his best judgment adopted everything new in practice which promised to be of service to his patients. Herein the overworked, the strenuous, the busy, and the prosperous man was a noble object lesson to every other man in the whole profession of medicine. When we have said of any man, as we can say of Sir Andrew Clark, that he was as thorough with his hundreds of patients as he had been with his units, and that he was as eager for new knowledge on his sixty-seventh birthday as he had been on his twenty-seventh, we have to that extent pictured a man who is not only rare in our generation, but has always been rare in the history of the world.

It would have been peculiarly appropriate, therefore, and encouraging to pure therapeutists, if the whole profession of medicine had spontaneously demanded for itself the honour of raising a memorial to so distinguished a therapeutist as Sir Andrew Clark. Medicine is not s pposed to be too generous to distinguished persons within its own ranks. But there are generous spirits within it, and these would have been glad if a spontaneous movement of the whole profession had enabled the affirmation to be made that the scientific medicine of to-day is more generous and appreciative than was the empirical and half-cultured physic of the previous centuries. Those spirits had indeed prepared a scheme whereby a true science of therapy, based on a true science of living pathology, might, so to speak, take a new departure in a permanent monument to the memory of the man who has just passed away. That scheme has for the moment failed of medical acceptance; but we earnestly hope for the sake of the highest progress of the therapeutic art, that it will be speedily revived.

The public and popular memorial which it has been proposed to raise has been inaugurated with the most complete and touching success. The Duke of Cambridge has been placed at the head of the movement, and the venerable Premier has given his warmest sympathy, and has initiated the subscription list on such a scale that a memorial really worthy of the occasion and the man will be speedily raised. The London Hospital is the institution with which the name of Sir Andrew Clark has been honourably associated for close upon half a century; it is to be the institution which is to hold his memorial for all future time. Nothing, we are sure, would better please the dead. There were two personalities in Sir Andrew Clark, as there inevitably are in every person who lives so near the centre and summit of modern civilisation as he did. There was the professional personality, restrained by the cramping and often purely artificial restraints of professional life; restraints loyally submitted to and never complained of. Behind this professional personality was the real man, quick, impulsive, enthusiastic, generous, a man to give himself away for an idea, and to strive with utmost urgency to carry all the world with him. It may be said that Sir Andrew Clark never did give himself away for an idea; but the answer, by one who took some pains to understand him, is that he did so give himself away; he gave himself away absolutely to the idea of high professional loyalty. Nobody saw more clearly than this man of clear mind and brilliant genius that professional loyalty demanded the sacrifice, not only of the least worthy, but sometimes of the more worthy self; that if he would satisfy the exacting demands of his more critical brethren he must sometimes sacrifice impulses which were not only honourable to himself, but which constitute some of the chief glories of man as man. We do not desire to emphasise this point, nor to enter into any controversy at the present time; but it is our hope that such injurious limitation of generous humanity by the purely artificial demands of a professional position may never again be imposed upon a president of the College of Physicians.

The poor are to benefit by the influence of Sir Andrew Clark's life whether his own profession be advantaged or not. If the profession be not also advantaged it alone will be responsible, and it alone will suffer. The greater world will not suffer, for it has long recognised, and now recognises more vividly than ever the truly worthy humanity of the man, a humanity which might have been so much greater and worthier if his natural impulses had been allowed unrestricted play. The London Hospital is to have a new wing—an "Andrew Clark Wing." This will be a truly great and a truly appropriate memorial. Sir Andrew lived to relieve pain, to cure his patients, and to make them happy. He was a born physician. At diagnosis he was excellent, but at treatment he was incomparable. It mattered not to him whether his patient were the Premier or the poorest man in East London. With all his intellect and with all his soul he threw himself upon the case, and was never satisfied until he had placed his patient in such a position that the utmost relief or the speediest cure should be the necessary result of his work. A new wing for the London Hospital has long been needed! A new wing! It would be more truly true to say a new hospital. In Whitechapel, where the London Hospital stands, a hospital is the most humane, the most truly benevolent, the most sorely-needed of all institutions. If Sir Andrew Clark could speak from his grave he would tell us that no work which we could do could possibly be so dear and so entirely satisfactory to him as the building of a new wing to his beloved hospital. Let us hope that the new wing may be built and opened with that promptitude of spontaneous generosity which, living, he would have felt so dear to his heart.

